# Gender, Stress, and Mental Health Among Older African Americans

**DOI:** 10.1093/geroni/igab046.183

**Published:** 2021-12-17

**Authors:** Christy Erving

**Affiliations:** Vanderbilt University, Nashville, Tennessee, United States

## Abstract

Across studies on social stress exposure on the mental health of older African Americans, most investigate singular stress exposures (discrimination). Furthermore, this research rarely assesses gender differences in the psychological effects of specific stress exposures. I use the National Survey of American of Life to assess: (1) gendered patterns of stress exposure among older African Americans; (2) gendered nuances in the individual, collective, and cumulative effects of stress exposure on mental health. I find gender patterns of stress exposure differed by type of stressor. Women and men shared some stress predictors of mental health (everyday discrimination). Other stress predictors were specific to women (health-related mobility challenges) or to men (perceived neighborhood crime). Study findings challenge gerontologists to consider how race-gender groups are at distinct risks for stressors that elicit poor mental health and provide a call for tailored strategies for improving the psychological health of African American women and men.

